# Supplementary material for: A robot for overground physical human-robot interaction experiments
Source: PLoS One. 2022 Nov 10;17(11):e0276980. doi: 10.1371/journal.pone.0276980 (PMC9648723; doi:10.1371/journal.pone.0276980)
Supplement: S1 Appendix — (PDF) [file pone.0276980.s001.pdf]

S1 Appendix. Interaction dynamics of the combined human arm-robot manipulator system: The interaction dynamics of a human arm is assumed to behave like a mass-spring-damper system, which involves passive, linear and time-invariant second-order dynamics. S1 Fig. shows a schematic representation of combined interaction dynamics of the robotic manipulator and the human arm right before the onset of perturbation (S1 Fig. A), and during the perturbation (S1 Fig. B).

The arm dynamics right before the onset of the perturbation can be represented as

$$f_{H0} = (m_H + m_{iH})\ \ddot{x}_0 + b_H\ \dot{x}_0 + k_H\ (x_0 - x_H) \tag{S1 Eq.}$$

where f_{H0} is the elastic restoring force of the arm right before the onset of perturbation. $\ddot{x}_0$ and $\dot{x}_0$ are the velocity and acceleration of the interaction handle at that instant, respectively.

The robotic manipulator is stiffness controlled with respect to the displacement from the workspace center (x_R). Hence, the force maintained by the stiffness controller right before the onset of perturbation is,

$$f_{R0} = k_R\ (x_0 - x_R) \tag{S2 Eq.}$$

At any instant before the perturbation onset, the force measured by force/torque sensor is equal to the elastic restoring force from the arm and the force maintained by the stiffness controller. Hence, force measured right before the onset of perturbation is

$$f_0 = f_{R0} = f_{H0}. \tag{S3 Eq.}$$

Substituting S1 Eq. in S3 Eq., we get

$$f_0 = (m_H + m_{iH})\ \ddot{x}_0 + b_H\ \dot{x}_0 + k_H\ (x_0 - x_H) \tag{S4 Eq.}$$

The stiffness controller is deactivated when the force perturbation f_{target} is applied. The interaction dynamics after the perturbation is,

$$f(t) = (m_H + m_{iH})\ \ddot{x}(t) + b_H\ \dot{x}(t) + k_H\ (x(t) - x_H) \tag{S5 Eq.}$$

Subtracting S4 Eq. from S5 Eq., we get

$$f(t) - f_0 = (m_H + m_{iH})\ (\ddot{x}(t) - \ddot{x}_0) + b_H\ (\dot{x}(t) - \dot{x}_0) + k_H\ (x(t) - x_0) \tag{S6 Eq.}$$

For our experiment, all the variables except the system parameters ($m_H + m_{iH}$, b_H, and k_H) are known. Hence, the system parameters can be estimated using linear regression.

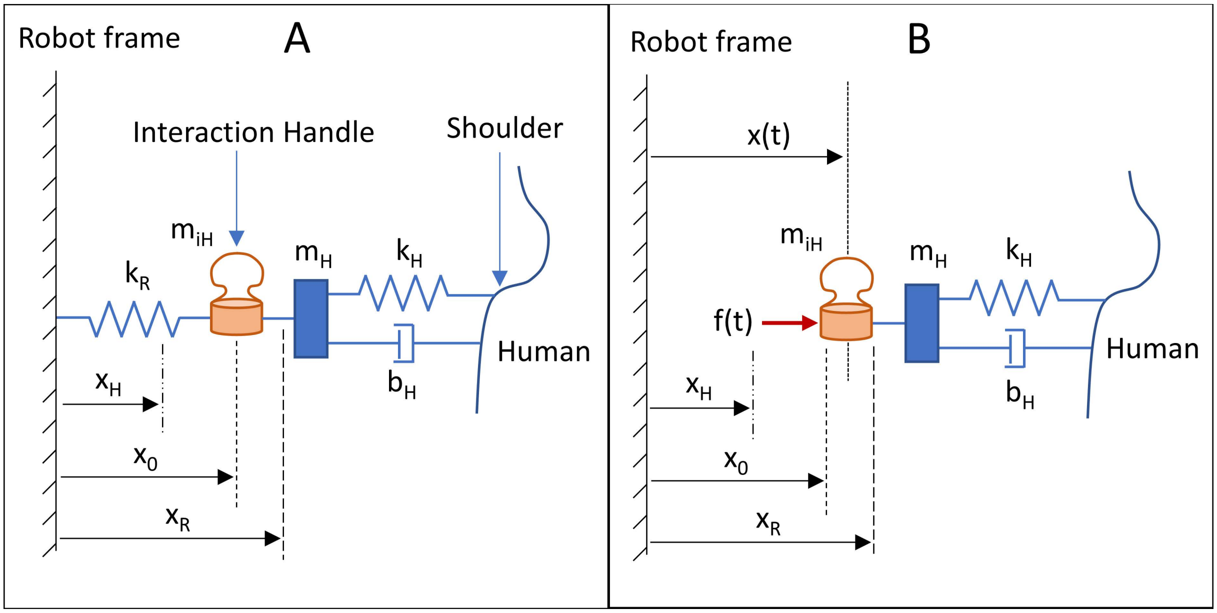

S1 Fig. The schematic representation of the interaction dynamics of the robot-human arm system during the experiment. Human arm model is characterized by mass (m_H), stiffness (k_H), and viscosity(b_H). The equilibrium point of the human arm is assumed to be at x_H. m_{iH} be the mass of the interaction handle. (A) At the instant right before the onset of the perturbation, stiffness controller with k_R stiffness is active whose equilibrium point is set at x_R. The interaction handle is at x_0 at this instant. (B) During the perturbation, the stiffness controller is replaced by interaction force controller (target force = f_{target}). The position of interaction handle at any instant during perturbation is represented by $x(t)$. The interaction force measured at that instant be $f(t)$. All the positions are represented with respect to the robot frame in this discussion.
