# Supplementary material for: A robot for overground physical human-robot interaction experiments
Source: PLoS One. 2022 Nov 10;17(11):e0276980. doi: 10.1371/journal.pone.0276980 (PMC9648723; doi:10.1371/journal.pone.0276980)
Supplement: Attachment — Submitted filename: response to reviewers.pdf [file pone.0276980.s002.pdf]

Reviewer #1: The choice of designing a robot for interaction experiments in motion is original, but some more details on the possible uses of this configuration would be useful.

We understand that there could be many questions about the design choices we made. Specifically for the configuration, we would ask the reviewer to examine our earlier publications on the design of the robot arm [30, 31].

The position of the hand is estimated based on the measurements of the motors, as well as the speed and acceleration. With regard to these last two quantities, has a simple numerical derivative been carried out or have filtering techniques been applied?

We simply took numerical derivatives from the filtered position data. No additional filtering was applied to the velocity data. As seen in Fig. 6, the velocity profile is reasonably smooth.

The position data was filtered with a zero-phase digital filter in both forward and reverse directions with a low-pass cut-off frequency of 40 Hz. This allowed rejecting the electrical noise interference (50 Hz or higher) and keeping the human movement data (5 Hz or lower). This information about filtering was added to the revision.

Is it possible to detect any traces of the sinusoidal movement of the shoulder in the experiments?

We did not see any obvious traces of shoulder movement in the hand movement or interaction force data collected by the robot. For example, in Fig. 4, the movement of the handle does not seem to show clear oscillatory movements around the center of the workspace. (note that the y-direction oscillation seen on the top right side of the figure is from an initial calibration step with intentional handle movement before the walking portion begins.)

We revised the manuscript to include our observation that the hand data did not seem to be affected by the shoulder movement.

By force perturbation is meant a rectangular impulse?

Yes, in our experiment, we used a rectangular pulse of one second as shown in Fig. 6. To make this more clear, we revised the manuscript to report the shape of the force profile.

The typical delay of the physiological response, even if mentioned by the authors, was not considered in the impedance model, a few more comments about this would help.

We agree that this point could be made more clearly. We revised the manuscript to include a concise version of our explanation below.

What we measured in this study is the impedance of the human arm at the instant of the perturbation onset over a short period of time (on average, 335.646 ms). Since the period is short, we assumed that the collective impedance of the system - including the instantaneous musculoskeletal effects and short-term neuromechanical effects - can be linearized into a second-order equation with lumped parameters. This approach has been incorporated in many prior works including [16, 19, 33, 34, 35]. In particular, our approach is similar to the method used in [19], which used short force perturbation and a linear lumped-parameter model for estimating the stiffness during dynamic conditions.

We did not intend to (nor is it possible to) identify the effects of neural delay itself. In the end, what affects the physical interaction is the impedance of the arm at specific instances.

Reviewer #2: The main contribution of this paper is the presentation of the design of a new mobile platform with a robotic arm attached to it, used for the measurement of the impedance of the human arm during walking. To this reviewer's knowledge, this is the first mobile platform used for this purpose, and the implementation is significant and of interest. This paper also provides the results of a preliminary experiment, where the same measurement of arm impedance is repeated in a number of conditions, i.e., under different visual conditions (eyes closed, eyes opened), and for different levels of perturbation and background stiffness.

This reviewer has a couple of major concerns:

1) Because this is a newly developed device, it would be good to validate the measurements of arm impedance vs. previous studies that quantified impedance in non-walking conditions. I think that an experiment in absence of walking (perhaps in a similar sitting position) would be useful to confirm the validity of the measurement of arm impedance obtained with this device. As the authors suggest, a lot of factors including the dynamics of the perturbing arm may affect the value of the measurements, but no validation is pursued for these target metrics.

Thank you for the suggestion. While the manuscript briefly mentioned that the arm stiffness values were comparable with those reported in seated experiments, the numeric values were not given. In this revision, we added the specific values of arm stiffness in the literature and also included a short discussion on the similarities and differences - especially on the possible effects of seated versus walking experiments.

2) I understand that the main focus of the paper is the presentation of the device and showing that it can measure arm impedance, and that the measurements are valid (see point above). However, there are a couple of concerns for the validation experiment pursued. I understand that the goal of the study is to understand how perturbation parameters affect the outcome, and

they end up showing that they don't (at least in a significant way), but what is the rationale for exploring the effect of visual condition? What was the hypothesis motivating the analysis, the rationale for forming such hypothesis, and the prediction?

Indeed, we had provided only a very short explanation of the inclusion of the vision condition (line 161). The vision condition was added to inform us about the design of future experiments whose protocol may or may not include vision (for example, leading a person on a curved path with or without vision). If so, we would like to know a priori if having or not having vision would interact with the other robot parameters, so that we may choose them accordingly in future experiments.

Our hypothesis was that, when measuring arm stiffness, the vision condition would interact with the level of background stiffness. When following a robot leader, one may want to know their position with respect to the leader (robot) at all times. Without vision and with low background stiffness, one would be less confident of the position of the leader and thus may subsequently modulate their arm stiffness. Our prior work suggested that low arm stiffness may help with increased sensitivity to small interaction forces [38]. If so, lowering arm stiffness would be beneficial for scenarios with increased uncertainty such as having no vision as well as low background stiffness. Our prediction was that, with no vision, the arm stiffness would be lower during low background stiffness trials compared to the arm stiffness during high background stiffness trials. But with vision, there would be little to no difference in arm stiffness between different background stiffness levels. Contrary to our prediction, the vision condition did not interact with any of the robot parameters and especially with the background stiffness, implying that at least within the provided robot parameter set, the availability of vision does not affect arm stiffness measurements.

As the reviewer correctly pointed out in their earlier comment, since arm stiffness measurement experiments were previously not conducted during walking, there were little to no comparable references to establish our rationales about any choices we made in our work. With our manuscript, however, we hope to provide the research community with the first exploratory measurements and their implications on overground pHRI experiment design.

The revisions for this comment were made across the manuscript. They can be found in 'Materials and Methods', 'Results', and in the 'Discussion' sections.

Also I have a concern about the statistical analysis used by the authors. You use here a full-factorial ANOVA, but subject should be considered a random effect rather than a fixed effect. A linear mixed model is more appropriate for this purpose given that the same participants are tested in multiple conditions. The study may not be powered to detect high-level interactions between the random effects and all combinations of the fixed effects, and these high-level terms may be removed, especially in absence of a strong rationale for how these high level terms would be interpreted.

First of all, thank you for this comment which led us to revisit our statistical analysis. We agree that there are two concerns with our initial analysis: using ‘subjects’ as a fixed effect, and including high-level interactions with possibly underpowered data without a strong rationale.

To remedy these in this revision, we used a linear mixed effects model where ‘subjects’ was considered as a random effect. This model included all possible interactions between the fixed effects (vision, interaction force magnitude, and level of background stiffness), but no random slopes representing interactions between the ‘subject’ and the fixed effects. We found that the variance due to the random effect was around 18.5% of the total variance in the data. Regarding the fixed effects, none of the main effects nor interactions was significant - consistent with the conclusion from our initial analysis with the 4-way ANOVA.

While it is certainly possible the effects of vision, interaction force, and background stiffness could vary between participants, a proper analysis of such variation would require many more participants, as the reviewer also noted.

We modified the corresponding paragraphs in Methods as well as in Results to reflect this change in the analysis.
